# Synthesis and Structure of the Double-Layered Sillén–Aurivillius Perovskite Oxychloride La_2.1_Bi_2.9_Ti_2_O_11_Cl as a Potential Photocatalyst for Stable Visible Light Solar Water Splitting

**DOI:** 10.1021/acs.inorgchem.3c00116

**Published:** 2023-04-20

**Authors:** Valérie Werner, Ulrich Aschauer, Günther J. Redhammer, Jürgen Schoiber, Gregor A. Zickler, Simone Pokrant

**Affiliations:** † Department of Chemistry and Physics of Materials, University of Salzburg, Jakob-Haringer-Str. 2A, 5020 Salzburg, Austria; ‡ Department of Chemistry, Biochemistry and Pharmaceutical Science, University of Bern, Freiestrasse 3, 3012 Bern, Switzerland

## Abstract

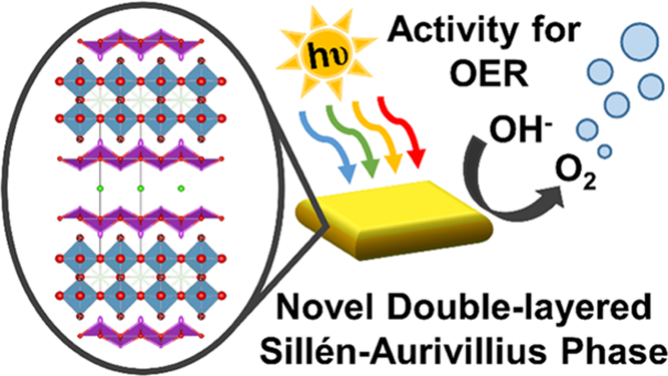

Exploring photocatalysts for solar water splitting is a relevant step toward sustainable hydrogen production. Sillén–Aurivillius-type compounds have proven to be a promising material class for photocatalytic and photoelectrochemical water splitting with the advantage of visible light activity coupled to enhanced stability because of their unique electronic structure. Especially, double- and multilayered Sillén–Aurivillius compounds [A_*n*–1_B*_n_*O_3*n*+1_][Bi_2_O_2_]_2_X*_m_*, with A and B being cations and X a halogen anion, offer a great variety in material composition and properties. Yet, research in this field is limited to only a few compounds, all of them containing mainly Ta^5+^ or Nb^5+^ as cations. This work takes advantage of the outstanding properties of Ti^4+^ demonstrated in the context of photocatalytic water splitting. A fully titanium-based oxychloride, La_2.1_Bi_2.9_Ti_2_O_11_Cl, with a double-layered Sillén–Aurivillius intergrowth structure is fabricated via a facile one-step solid-state synthesis. A detailed crystal structure analysis is performed via powder X-ray diffraction and correlated to density functional theory calculations, providing a detailed understanding of the site occupancies in the unit cell. The chemical composition and the morphology are studied using scanning and transmission electron microscopy together with energy-dispersive X-ray analysis. The capability of the compound to absorb visible light is demonstrated by UV–vis spectroscopy and analyzed by electronic structure calculations. The activity toward the hydrogen and the oxygen evolution reaction is evaluated by measuring anodic and cathodic photocurrent densities, oxygen evolution rates, and incident-current-to-photon efficiencies. Thanks to the incorporation of Ti^4+^, this Sillén–Aurivillius-type compound enables best-in-class photoelectrochemical water splitting performance at the oxygen evolution side under visible light irradiation. Thus, this work highlights the potential of Ti-containing Sillén–Aurivillius-type compounds as stable photocatalysts for visible light-driven solar water splitting.

## Abstract 2

Exploring photocatalysts for solar water splitting is a relevant measure to take toward sustainable hydrogen production. In this work, a fully titanium-based oxychloride, La_2.1_Bi_2.9_Ti_2_O_11_Cl, with a double-layered Sillén−Aurivillius intergrowth structure is fabricated via a facile one-step solid-state synthesis. A detailed crystal structure analysis is performed via powder X-ray diffraction and correlated to density functional theory calculations, followed by the demonstration of its photocatalytic activity for visible light-driven water oxidation.

## Introduction

Photocatalytic and photoelectrochemical water splitting are potential pathways to provide renewable hydrogen.^1,2^ In this context, the development of novel, stable materials showing photocatalytic activity toward the water splitting reaction is a crucial step. A variety of inorganic semiconducting oxides were proven to demonstrate photocatalytic activity for the water splitting reaction.^3−5^ TiO_2_ is one of the most studied compounds within this class, exhibiting excellent photocatalytic properties.^6,7^ However, similar to TiO_2_, many oxides cannot absorb visible light since they have large band gaps due to the low-lying O 2p orbitals that form the valence band edge (VBE). Narrowing the band gap by lifting the VBE via N, S, or X (X = I, Br, Cl) incorporation as in (oxy)nitrides, (oxy)sulfides, and oxyhalides leads to excellent visible light absorption and consequently to improved light harvesting properties.^8,9^ However, a material with an oxygen-dominated VBE is inherently chemically more stable in aqueous electrolytes than nitrogen-, sulfur-, or halide-containing compounds since an orbital-energetic driving force exists to exchange these elements with oxygen originating from water.^10^ Instead of modifying the VBE, shifting the conduction band edge (CBE), which is sensitive to the cations, is a potential alternative, promising improved stability.

Recently, bismuth-based layered oxides have increasingly attracted attention as photocatalysts for solar water splitting due to their unique layered crystal structure, suitable electronic band structure, and diverse composition.^11^ In addition, other application areas, such as oxide ion conductors in electrochemistry, have been explored.^12^ Structurally, these materials can be categorized as Sillén, Aurivillius, and Sillén–Aurivillius-type compounds. Sillén-type compounds, *e*.*g*., BiOX, are formed by [Bi_2_O_2_]^2+^ layers intergrown by one (*m* = 1) or several (*m* > 1) halogen X*_m_* layers. In BiOX with X = Cl, I, and Br, the VBE is raised due to the hybridization of the Cl 3p, Br 4p, I 5p, and O 2p orbitals.^13−15^ As a consequence, the ability to absorb visible light varies with the type of halogen ion. With decreasing electronegativity of the halogen ion, the band gap narrows from 3.42 eV (BiOCl) to 1.84 eV (BiOI), enabling efficient light harvesting.^16^ Aurivillius-type structures, [A_*n*–1_B*_n_*O_3*n*+1_][Bi_2_O_2_], consist of alternating [Bi_2_O_2_]^2+^ layers and perovskite slabs [A_*n*–1_B*_n_*O_3*n*+1_]^2–^, where *n* is the number of octahedral layers. They can provide suitable electronic structures for the absorption of visible light and offer the possibility to modify the band structure by A- or B-site substitution.^17,18^ For example, in *n* = 3 compounds with A = Bi^3+^ and B = Ti^4+^, i.e., Bi_4_Ti_3_O_12_, the valence band is mainly formed by O 2p states and the conduction band is predominantly formed by Ti 3d with minor contributions of Bi 6p orbitals, resulting in a narrower band gap (2.5–2.8 eV) compared to TiO_2_ (3.2–3.4 eV).^19^ The third class, Sillén–Aurivillius-type intergrowth structures, [A_*n*–1_B*_n_*O_3*n*+1_][Bi_2_O_2_]_2_X*_m_*, contain [Bi_2_O_2_]^2+^ layers that are interleaved with both halogen layers, X*_m_*, and perovskite slabs, [A_*n*–1_B*_n_*O_3*n*+1_]^2–^, and show light absorption in the visible range with band gaps between 2.39 and 2.86 eV.^13,20−22^ In contrast to Sillén-type oxyhalides (e.g., BiOCl) and other mixed anion materials (e.g., oxynitrides, oxysulfides) where the VBE mainly consists of Cl 3p, N 2p, or S 3p orbitals, the VBE of Sillén–Aurivillius-type oxyhalides is mainly dominated by O 2p orbitals, leading to enhanced resistance toward oxidative degradation during water splitting.^13,16^

The origin of the peculiar valence band structure of these materials is somewhat controversial based on both theoretical calculations and experimental investigations.^13,16,21^ Kato et al. and Fujito et al. suggest that the valence band of the material contains highly dispersive O 2p orbitals that form the VBE rather than the halogen anion orbitals,^13,16^ whereas the study of Kunioku et al. proposes that the VBE of Bi_4_BO_8_X (B = Nb, Ta; X = Cl, Br) consists of Bi 6s, Bi 6p, and O 2p orbitals and that the elevated VBE can be partly explained by the hybridization of Bi 6s and O 2p orbitals.^21^ The CBE of these compounds, however, is consistently described in the literature as predominantly formed by Bi 6p orbitals.^13,21^ Due to this distinct electronic band structure and suitable light absorption ability, the bismuth-based layered Sillén–Aurivillius-type perovskite oxyhalides are reported as candidates for efficient and stable water splitting under visible light, which is especially relevant for practical solar water splitting.^13,20,22^

Single-layered Sillén–Aurivillius-type oxyhalides, [A_*n*–1_B*_n_*O_3*n*+1_][Bi_2_O_2_]_2_X*_m_* with *n* = 1, (A = Bi; B = Ta, X = Cl, Br, I; or B = Nb, X = Cl, Br) and *m* = 1, are the simplest and most explored Sillén–Aurivillius-type oxyhalides and exhibit photocatalytic and ferroelectric properties.^13,23,24^ As expected, the influence of the halide ion on the band gap of these compounds is minor. For example, Bi_4_NbO_8_Cl exhibits a band gap of 2.4 eV and Bi_4_NbO_8_Br of 2.5 eV. This slight change is reversed and less pronounced than in the corresponding Sillén-type compounds where a change from 3.4 eV for BiOCl to 2.6 eV for BiOBr is observed.^16^ A similar trend to that for the Nb-containing Sillén–Aurivillius-type oxyhalides is obtained for Ta-based Bi_4_TaO_8_X compounds, where the band gap changes only slightly from 2.50 eV for Bi_4_TaO_8_Cl to 2.55 eV for Bi_4_TaO_8_Br.^21^ In addition, the cations on the A- and the B-sites can be altered, influencing the material properties. In previously reported single-layered Sillén–Aurivillius-type compounds, the B-site is primarily occupied by either niobium or tantalum with few exceptions such as, for example, vanadium.^13,21,23,25−27^ Recently, Kulczny et al. reported that partial substitution of Bi^3+^ in Bi_4_NbO_8_Cl with bi- and tetravalent cations (Sr^2+^, Ba^2+^, Sn^4+^) leads to oxygen vacancies or interstitial oxygen respectively allowing to tune the oxide ion conductivity.^12^ Moreover, Wei et al. reported enhanced photocatalytic activity toward the hydrogen evolution reaction for partial yttrium substitution in Bi_4_NbO_8_Cl (i.e., Bi_4–*x*_Y*_x_*NbO_8_Cl with *x* = 0, 1, 1.33, 2, 2.67, 3).^28^ Even though the material properties are altered to an extent by varying the halide anions and A- and B-cations, the parameter space to further expand the compositional versatility of *n* = 1 Sillén–Aurivillius-type oxyhalides is limited. Hence, the synthesis of double- and multilayered Sillén–Aurivillius-type oxyhalides, [A_*n*–1_B*_n_*O_3*n*+1_][Bi_2_O_2_]_2_X*_m_*, (*n* ≥ 2 and *m* = 1), offers a greater variety of compounds.^19,22,29,30^ A series of double-layered compounds A′_1+*x*_A_4–*x*_B_2_O_11_X (A = Bi; A′ = Pb, Ba, Sr; B = Ta, Nb; X = Cl, Br, I) have already been proposed in recent reports.^16,20,31−33^ Also in these compounds, varying the halide ions tunes the band gap and enhances the photocatalytic activity toward the oxygen evolution reaction as reported by Ogawa et al. for Ba_2_Bi_3_Nb_2_O_11_X (X = Cl, Br, I).^32^ As for single-layered (*n* = 1) compounds, most of the studies on double-layered (*n* = 2) Sillén–Aurivillius-type oxyhalides refer to Nb- and Ta-based compounds. In A′_2_Bi_3_B_2_O_11_Cl compounds (A′ = Ba, Sr; B = Ta, Nb), partial substitution of the pentavalent B-cation with Ti^4+^ was reported to enhance the photoelectrochemical performance in combination with Bi^3+^ cosubstitution at the A′-site. The introduction of Ti^4+^ on the B-site resulted in a Ti 3d contribution to the conduction band in addition to Bi 6p and Ta 5d orbitals.^20^ The authors showed that the increased Bi-content leads to a further narrowing of the band gap improving the visible light absorption properties of these compounds. They propose that this is the main reason for the improvement of the photocatalytic properties.^20^ Considering that Ti^4+^-containing perovskite oxides like SrTiO_3_ are known for their excellent photocatalytic properties but also for being limited to UV light absorption,^34,35^ double-layered Sillén–Aurivillius-type oxyhalides provide an interesting structural frame to implement the photocatalytically very active [TiO_3_]^2–^ unit. However, to the best of our knowledge, so far there have been no reports on double-layered Sillén–Aurivillius compounds exclusively based on Ti^4+^.

In this work, a novel double-layered Sillén–Aurivillius-type structure containing exclusively Ti^4+^ at the B-site is proposed. In order to maintain charge neutrality, La^3+^ cations are cosubstituted on the A′-site since La^3+^ is known to form stable perovskite oxides with Ti.^36,37^ First, a facile one-step flux-assisted solid-state synthesis for the compound A′_2±δ_A_3±δ_B_2_O_11_Cl, with A′ = La, A = Bi, and B = Ti, is developed. The crystal structure of the novel compound is determined by combining experimental and theoretical methodologies, in addition to morphology and composition. The optical properties are determined experimentally and compared to electronic structure calculations. Finally, the photoelectrochemical and photocatalytic activity of the compound toward the oxygen evolution reaction is demonstrated.

## Materials and Methods

### Materials

La_2_O_3_ (99.9%), TiO_2_ (anatase, 99.9%), BiOCl (98%), and Na_2_SO_4_ (99%) are purchased from Sigma-Aldrich. Bi_2_O_3_ (99.9%) and acetone (pro analysis) are purchased from Acros Organics and Supelco, respectively. NaCl (99%), KCl (99.5%), and iodine (99%) are purchased from VWR Chemicals.

### Synthesis

The double-layered compound A′_2±δ_A_3±δ_B_2_O_11_Cl, with A′ = La, A = Bi, and B = Ti, is prepared via a flux-assisted solid-state synthesis. Prior to the synthesis, La_2_O_3_ is dried at 500 °C for 4 h. For the one-step solid-state synthesis, the precursor compounds La_2_O_3_, Bi_2_O_3_, TiO_2_, and BiOCl are mixed in the stoichiometric ratio of 1:1:2:1 after adding a NaCl/KCl mixture as flux (molar ratio: 1:1) the compounds are thoroughly mixed in an agate mortar for 30 min. The amount of flux is set as a 100 eq. of the amount of TiO_2_. The mixture is then transferred into Al_2_O_3_ crucibles and kept at 780, 800, or 820 °C for 5, 10, or 20 h, followed by cooling to room temperature in an electric furnace (Nabertherm, P330). Afterward, the powder is dispersed in water, filtrated under suction, washed vigorously with ultrapure water (Millipore, 15 MΩ cm), and dried at 80 °C for 5 h.

### Structural, Compositional, and Morphological Characterization

Phase identification and indexing (unit cell parameter determination) are performed using data collected to *d*_min_ values of 0.87 on a Bruker D8 Advance diffractometer (Cu Kα_1,2_ radiation, step size 0.01°, solid-state LynxEye detector). Zero-background single-crystal silicon sample holders are covered with a thin layer of the synthesized compound. Additional data are collected at the European Synchrotron Radiation Facility (ESRF) at beamline SNBL-BM01 with a monochromatic high-brilliant X-ray beam with λ = 0.6053 Å. The diffractometer is based on a Pilatus2M detector,^38^ and two different data sets (high resolution with *d*_min_ = 0.83 Å and high *q*-space with *d*_min_ = 0.56 Å) are recorded. For measurements, the sample is filled into a 0.2 mm glass capillary, which was rotated throughout 200°, thus also minimizing preferred orientation effects. Data processing is performed with SNBL software Bubbles.^38^

XRD powder patterns are first indexed and refined via the Rietveld method using software TOPAS (Version 6.0, Bruker AXS Inc., WI).^39^ Additional tests using JANA2006 and the built in Superflip software are conducted.^40^ The crystal structure is resolved using a combination of locating the heavy atoms localization using Superflip, model refinement, Fullprof, and Fourier as well as difference Fourier analysis (GFourier as inbuilt in the Fullprof-Suite of programs).^41^ Final structure refinements of the synchrotron data are done using the Fullprof-Suite. The background is determined using linear interpolation between a set of background points with refinable heights, the peak shape is modeled using the Thompson–Cox–Hastings pseudo-Voigt function. Absorption correction has been applied. The visualization of the crystal structure is performed using VESTA.^42^

Selected-area electron diffraction (SAED) patterns and high-angle annular dark-field (HAADF) z-contrast scanning transmission electron microscopy (STEM) images are acquired using a JEOL F200 TEM/STEM equipped with a cold field emission gun and operated at 200 kV with a TVIPS F216 2k using a 2k CMOS TEM camera. A large windowless JEOL Centurio EDX detector (100 mm^2^, 0.97 srad, energy resolution < 133 eV) is used for signal detection. TEM grids are prepared by suspending the synthesized powder in dry ethanol, followed by deposition on a copper grid with a holey carbon thin film. EDX intensity maps and spectra were acquired with a typical beam current of 0.3 nA and a beam diameter of 0.23 nm. The EDX maps were obtained by integrating the counts over a specific transition: La L_α_ line for La (integration: 4.59–4.79 keV), O K_α_ line for O (integration: 0.46–0.59 keV), Ti K_α_ line for Ti (integration: 4.37–4.56 keV), Bi M_α_ line for Bi (integration: 2.31–2.53 keV), and Cl K_α_ line for Cl (integration: 2.59–2.73 keV). The edges were chosen such that the signal-to-noise ratio allowed quantitative evaluation while minimizing absorption.

Images of the carbon-coated samples are acquired with a Zeiss Ultra Plus 55 scanning electron microscope (SEM) using an in-lens secondary electron detector, a working distance of 4 mm and an acceleration voltage of 3 kV. The particle size range is determined with the aid of evaluation software SmartTiff (Version 3, Carl Zeiss Microscopy) based on 154 particles. The particle thickness is determined similarly based on 20 particles. Energy-dispersive X-ray spectroscopy is conducted by using an EDX detector (50 mm^2^ silicon drift detector) from Oxford instruments, a working distance of 9 mm, a 30 μm aperture, and an accelerating voltage of 15 kV.

The specific surface area is acquired via nitrogen physisorption. The acquisition of physisorption isotherms is performed with a Micromeritics ASAP 2420 sorption apparatus at −196 °C using a sample mass of 2 g. The sample undergoes a degassing step at 150 °C for 2 h prior to the measurement. The Brunauer–Emmett–Teller (BET) method is applied to determine the specific surface areas (m^2^ g^–1^) using the adsorption curve.^43^

### Optical Characterization

UV–vis diffuse reflectance data are obtained using an UV–vis–NIR spectrophotometer (PerkinElmer, Lambda 1050) over a spectral range of 200–900 nm (step size 2 nm) and BaSO_4_ as a reference. Using the Kubelka–Munk equation *F*(*R*) = ((1 – *R*)^2^)/(2·*R*), where *R* is the absolute reflectance of the sample, the band gap is estimated by extrapolation.^44^ The error of this method is estimated to be ±0.05 eV.^45^

### Photoelectrochemical Measurements

Particle-based photoanodes are fabricated by electrophoretic deposition as previously reported.^46^ Powder suspensions are prepared containing 45.3 mg of the synthesized compounds, 12.5 mg of iodine, and 62 mL of acetone. For the deposition, two substrates (fluorine-doped tin oxide glass slides, 15 Ω sq^–1^, XOP Glass) are immersed into the suspension with a separation of 6.5 mm and the conducting sides facing each other. A voltage of 20 V is applied for 210 s. After each minute, the solution is stirred for 10 s at 450 rpm. Post-modification of the particle-based electrodes consisted of necking with TiO_2_, which is conducted via dip-coating.^47^ Photoelectrochemical measurements are carried out in a three-electrode configuration using the prepared particle-based photoelectrode as a working electrode, a Pt-metal wire as a counter electrode, and a Ag/AgCl reference electrode (stored in 3 M KCl). For the measurement, an aqueous solution of 0.1 M Na_2_SO_4_ with a pH of 2.26 (adjusted by adding H_2_SO_4_) is prepared and used as an electrolyte. For the investigation of the influence of the pH value of the electrolyte, additionally 0.1 M Na_2_SO_4_ solutions with a pH value of 7.00 and 13.45 (adjusted by adding NaOH) are prepared. A 300 W Xe lamp (Lot Oriel) equipped with an AM 1.5G filter with and without an UV-cutoff filter is used as a light source and calibrated to an intensity of 100 mW cm^–2^ with the aid of a Si photodiode. Linear scan voltammetry measurements (cathodic and anodic sweep) in a potential range of 0–1.23 V vs RHE are conducted with a scan rate of 0.01 V s^–1^. Chronoamperometric measurements at a potential of 1.2 V vs RHE for 900 s are performed using a VersaSTAT 4 potentiostat. The illuminated circular area has a radius of 4.25 mm and back illumination through which the substrate is applied. The measurements are conducted under alternating illumination (shutter is opened and closed repetitively). At least two electrodes are measured for each data point. The thickness of the particle-based electrodes is determined using a profilometer DektakXT (Bruker) equipped with a 2 μm stylus. For each electrode, the thickness is determined over a distance of 5 mm at three positions.

### Incident-Photon-to-Current Efficiency

Incident-photon-to-current conversion efficiencies (IPCEs) are determined by using a 300 W Xe light source (Lot Oriel) equipped with band pass filters (Andover Corporation) with central wavelengths of 400, 420, 460, 500, 550, 600, 650, and 700 nm and a full width at half-maximum of 10 nm. Chronoamperometric measurements under back illumination are performed in a three-electrode configuration as described before. A potential of 1.2 V versus RHE is applied, and 0.1 M Na_2_SO_4_ with a pH of 2.26 (adjusted by adding H_2_SO_4_) is used as an aqueous electrolyte. IPCE is calculated via the equation IPCE% = [(*j*·*h*·*c*)/(*P*_mono_·λ·*e*)] × 100% and is based on measurements of two photoelectrodes, where *h* is Planck’s constant, *c* is the speed of light, *e* is the electric charge of an electron, λ is the wavelength of the corresponding band pass filter, and *P*_mono_ is the light intensity, which is determined using a calibrated Si photodiode. The samples are illuminated alternately using a shutter, which is open for 10 s and closed for 3 s. The photocurrent density, *j*, is determined by taking the average value of four illumination periods.

### Photocatalytic Measurements

For photocatalytic measurements, 40 mg of the oxyhalide material are suspended in 5 mM FeCl_3_ (pH 2, adjusted by adding HCl). After repeated evacuation of the gas-tight reaction chamber followed by and flushing it with Ar, the powder suspension is irradiated with standard illumination (300 W, LoT Oriel, 100 mW cm^–2^) for 5 h while stirring continuously at 350 rpm. The evolved gases are quantified hourly using gas chromatography (Micro GC Fusion, type: F0Q400UC2, INFICON).

### Density Functional Theory Calculations

Density functional theory (DFT) calculations are performed at the PBEsol level of theory with the Vienna Ab initio Simulation Package (VASP), expanding wavefunctions in planewaves with a kinetic energy cutoff of 600 eV.^48−51^ Electron–core interactions are described using the PAW method, treating La (5s, 5p, 5d, 6s), Bi (5d, 6s, 6p), Ti (3s, 3p, 3d, 4s), Cl (3s, 3p), and O (2s, 2p) as valence electrons.^52,53^ For the 3.84 Å × 3.84 Å × 17.85 Å *P*4/*mmm* unit cell, reciprocal space is sampled using a 8 × 8 × 2 mesh, with appropriately reduced mesh dimensions for supercells. Structures are relaxed until forces converged below 10^–3^ eV Å^–1^ and stresses below 5 × 10^–5^ eV Å^–3^. Phonon modes are determined within the harmonic approximation using the Phonopy package.^54^

## Results and Discussion

### Synthesis, Structure, and Composition

In this work, a one-step flux-assisted solid-state synthesis method was developed for the double-layered (*n* = 2) Sillén–Aurivillius-type oxyhalide, A′_2±δ_A_3±δ_B_2_O_11_Cl, with A′ = La, A = Bi, and B = Ti, containing exclusively Ti^4+^ at the B-site. Flux-assisted solid-state syntheses of bismuth oxyhalides have shown an enhanced photoactivity since unfavorable halogen defects are avoided and a higher crystallinity is promoted when halogen-containing salts are used as flux.^55,56^ High crystallinity is considered as an important prerequisite for good photocatalytic properties.^3^ Additionally, due to the increased ion mobility during the synthesis, lower synthesis temperatures and shorter reaction times are possible.^11^ Reported synthesis procedures for double-layered Sillén–Aurivillius-type compounds vary widely.^20,30,31,33^ Double-layered Sillén–Aurivillius-type oxyhalides such as Sr_2_Bi_3_Ta_2_O_11_Cl or Ba_2_Bi_3_Ta_2_O_11_Cl require high temperatures and long reactions times (950 °C for 35 h) or cannot be formed at all by using a solid-state synthesis approach.^20,29^ Hence, a two-step method is often used to synthesize single- and double-layered Sillén–Aurivillius-type oxyhalides.^12,29,30^ Therefore, the facile one-step flux-assisted solid-state synthesis approach developed in this work (820 °C, 20 h) is advantageous in comparison to literature procedures for reasons of simplicity and lower energy cost.

**Figure 1:**
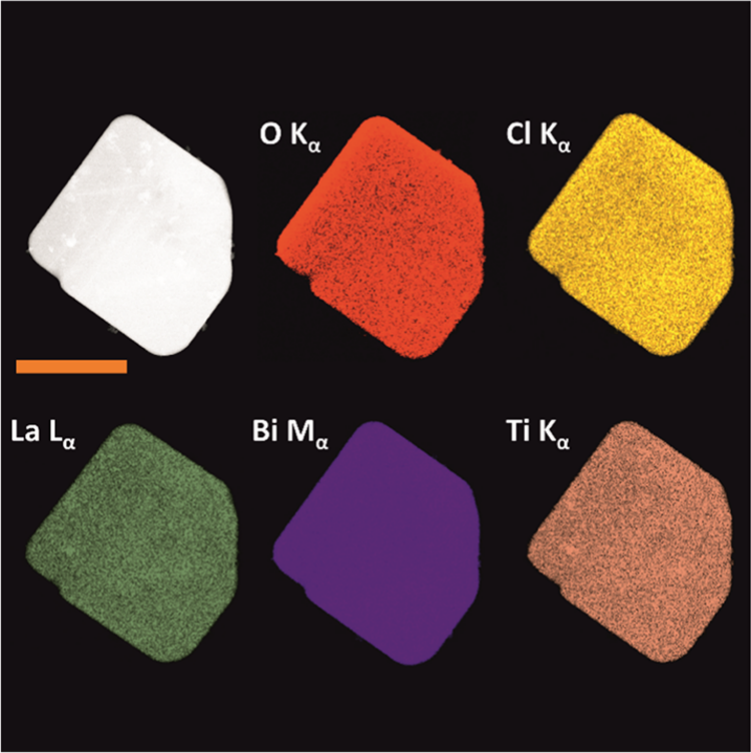
HAADF image and STEM-EDX intensity maps of an A′_2±δ_A_3±δ_B_2_O_11_Cl, with A′ = La, A = Bi, and B = Ti, particle. The scale bar corresponds to 1 μm.

The composition of the compound A′_2±δ_A_3±δ_B_2_O_11_Cl, with A′ = La, A = Bi, and B = Ti, was investigated by a combination of STEM and SEM-EDX experiments. STEM-EDX intensity maps of a representative particle (Figure 1, scale bar corresponds to 1 μm) and SEM-EDX maps of several particles (Figure S1) show that the elemental distribution is homogeneous, which is an indication for the absence of additional phases. By analyzing the SEM-EDX maps quantitatively, atomic ratios are determined (Table S1) and compared to theoretical values based on the stoichiometric ratios used for synthesis. The atomic La/Ti ratio is approximately 1.09, whereas the Bi/Ti ratio is about 1.41, indicating that the composition of the compound slightly differs from the theoretical values of 1.00 and 1.50, respectively. The Cl/Bi ratio is with 0.29 slightly lower than the target value of 0.33 but within the range of the expected accuracy of EDX measurements. Overall, the theoretical composition of the compound is roughly confirmed by the atomic ratios determined by EDX results.

**Figure 2:**
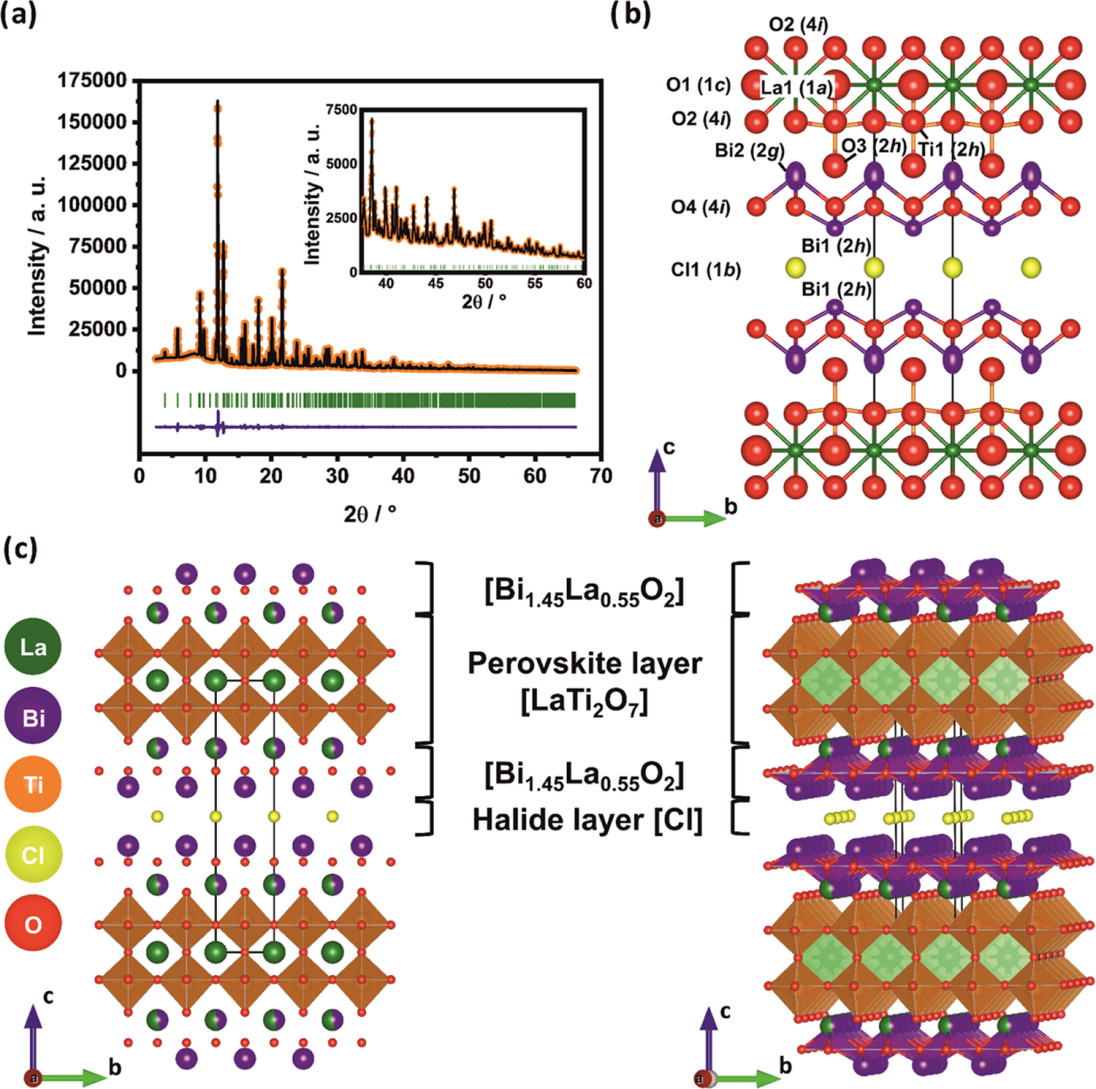
Refined high-*q* synchrotron XRD diffraction pattern and structure refinement fit (a) and derived crystal structure of La_2.1_Bi_2.9_Ti_2_O_11_Cl with cations drawn with anisotropic atomic displacement parameters at a 95% probability level (b). The inset in panel (a) displays the high-angle region at an enlarged *y*-scale. Crystal structure of La_2.1_Bi_2.9_Ti_2_O_11_Cl with mixed occupation at the 2g position (c). The intergrowth layers are assigned. Yellow spheres denote Cl atoms, red spheres denote O atoms, purple spheres denote Bi atoms, green spheres denote La atoms, and orange spheres denote Ti atoms.

Powder XRD patterns are used to determine the crystal structure (see Figures 2a and S2) assuming that the compound is phase pure as suggested by EDX analysis. The diffraction pattern can be indexed based on a tetragonal unit cell. Autoindexing using TOPAS proposes two different unit cells, one with *a* = 5.4625 Å and *c* = 17.9926 Å and a tentative space group *P*4/*nbm* and a second one with *a* = 3.8626 Å and *c* = 17.9924 and a *P*4/*mmm* space group symmetry. The two lattice parameters *a* are related to each other via a √2 relation, i.e., the *a*_1_ and *a*_2_ parameters of the larger cell correspond to the diagonals in the *a*_1_ and *a*_2_ planes of the smaller one. Indexing with ITO and TREOR, as implemented in the FULLPROF-Suite, uniquely gives the smaller cell with a space group symmetry *P*4/*mmm* as a solution of autoindexing, thus in accordance with the general praxis to choose the smallest possible cell, the *a* = 3.8626 Å and *c* = 17.9924 cell is used for further considerations. Additional tests using JANA2006 and built in Superflip software confirm the *P*4/*mmm* space group symmetry.^40^ High-resolution synchrotron diffraction data give no evidence of asymmetric line broadening or weak superstructure reflections, thus confirming the chosen tetragonal unit cell based on laboratory X-ray diffraction data.

**Figure 3:**
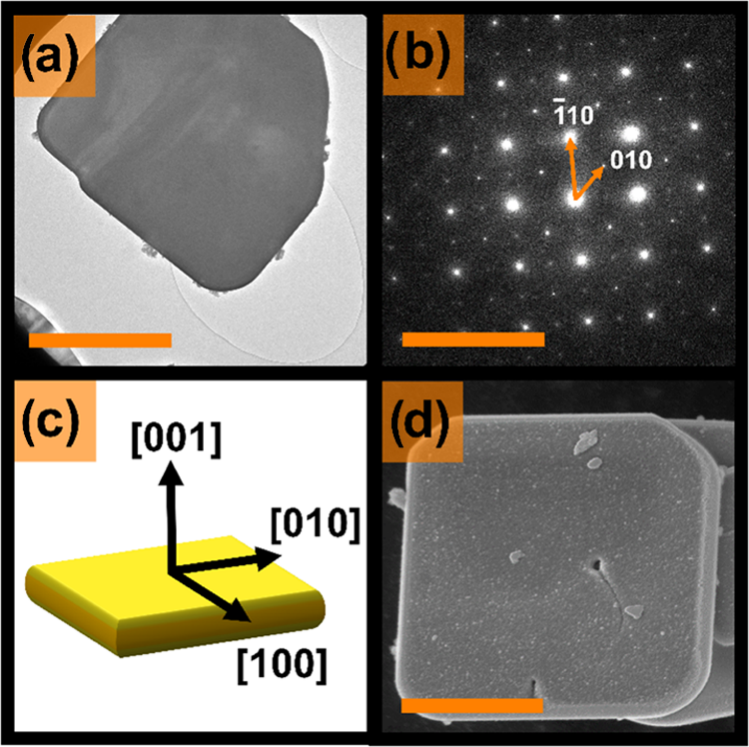
TEM image (a), corresponding SAED pattern (b), schematic crystal orientation (c), and SEM image (d), of a La_2.1_Bi_2.9_Ti_2_O_11_Cl particle. The scale bars are set to 1 μm (a), 10 nm^–1^(b), and 1 μm (d).

Comparing to TEM diffraction (Figure 3a (scale bar 1 μm) and Figure 3b (scale bar 10 nm^–1^) and Figure S3) the SAED pattern can be assigned to the tetragonal *P*4/*mmm* structure (ICSD-196066) assuming the [001] viewing direction.^31^ Additional weak reflexes are visible in [110] at 1/2 [110] (Figure S2), which could come from multiple scattering events in this thick sample. However, since these reflections might also be of kinematic nature since they are not affected by tilting (Figure S2), an interpretation of these diffraction spots as partial order in the ⟨110⟩ direction seems to be a possibility as well. Combining XRD and TEM results, the structure is described best by the smallest possible unit cell with *a* = 3.8626 Å and *c* = 17.9924 Å. With this cell and the proposed space group symmetry, all observed Bragg peaks of the XRD pattern can be indexed using Le Bail fits within TOPAS software. Moreover, this *P*4/*mmm* unit cell is similar to the ones of other Aurivillius- and Sillén–Aurivillius-type compounds, namely, Sr_2_Bi_3_Nb_2_O_11_Cl,^31^ Cu_0.6_Ba_2_Ca_3_Cu_4_O_10.8_,^57^ or Ba_2_Ca_3_(Cu_0.68_C_0.32_)Cu_4_O_11.06_^58^ and Ba_2_Bi_2_Nb_2_O_11_X (X = Cl, Br, I) as reported by Ogawa et al.^32^

Structure solution yields four different, strong Fourier peaks, corresponding to the heavy atoms in the crystal structure. Site occupation refinements show that the 1a-site (0 0 0 for *x y z* fractional atomic coordinates) is fully occupied by La^3+^ (La1), while Ti^4+^ fully occupies the 2h position (Ti1 at 1/2 1/2 0.116). Bi^3+^ is found with full occupation at the second 2h position (Bi1 at 1/2 1/2 0.392) as well as on the 2g position (Bi2 at 0 0 0.249). This Bi2 position shows a mixed occupation with La^3+^ and Bi^3+^. This is derived from a distinct underpopulation of this site when assigning only Bi^3+^ to it and allowing to refine the electron density freely. The reverse is observed, when positioning only La^3+^ to this site. Assuming full occupation with only one of the two atoms significantly worsens the reliability factors of the refinement (Table S2). A mixed occupation refinement with Bi^3+^ and La^3+^ at this Bi2-site (assuming full occupation) gives 0.55 La^3+^ and 0.45 Bi^3+^, slightly changing with data resolution.

A further, distinctly smaller Fourier peak is found at position 1b (0 0 1/2) and is assigned to Cl^–^. In subsequent refinement cycles, four nonequivalent oxygen atom positions can be identified, located at the special Wyckoff positions 1c, 4i, 2h, and 4i for the O1, O2, O3, and O4 oxygen atoms, respectively. In free refinements of site occupation factors, values close to full occupation with reliable isotropic atomic displacement parameters (ADPs) were obtained, and therefore, in final refinements, the site occupation numbers are fixed to full occupation. High-*q*-space synchrotron data allow the refinement of anisotropic ADPs for all cations (Figure 2b) while keeping the anions isotropic. The La1-, Ti1-, and Bi1-sites exhibit almost isotropic ADPs, while for the Bi2 position, more elongated anisotropic ADPs are observed. This can be explained by a slight positional disorder due to the mixed occupation of this site by La^3+^ and Bi^3+^.

It is evident, that especially the O1- and O2-sites, bonded to the La1-site, exhibit larger isotropic ADPs than the O3 and O4 oxygen atom positions. This may reflect a weaker bond strength due to higher coordination (see below) and larger bond lengths around La1 but may also hint to structural instabilities associated within the oxygen coordination of La^3+^ within the perovskite layer. For the compounds Ca_1.25_Sr_0.75_Bi_3_Nb_2_O_11_Cl, BaCaBi_3_Nb_2_O_11_Br, and Sr_2_Bi_3_Nb_2_O_11_Cl, Charkin et al. observed similar behavior and explained it by a positional disorder and a shift of the O1 and O2 oxygen atom positions from 1c to 2h and from 4i to general 8*s* position, respectively.^31^ A correct description of such a structural instability (and possible also the positional disorder at Bi2) would require the reduction of symmetry to the space group *P*1 and a doubling of the cell parameters along the *a* and *b* directions (see DFT calculations below). Even with the high-*q* synchrotron data, it is not possible to get stable refinements with such a model. The O4 oxygen atom, bonded to Bi^3+^ and Bi^3+^/La^3+^ cations at 2h- and 2g-sites, respectively, depicts the smallest atomic displacement, which might be due to more rigid bonding than for O1 and O2 oxygen atoms (see Figure 2b).

Figure 2c gives a polyhedral representation of the structure of the title compound. La^3+^ atoms in the perovskite layer are 12-fold coordinated by the O1 and O2 oxygen atoms, thereby forming a cubic-octahedral coordination polyhedron with La–O bond lengths ranging between 2.648(9) and 2.7315(6) Å. Ti^4+^ cations are octahedrally coordinated by the O1, O2, and O3 oxygen atoms with Ti–O bond lengths of 2.078(4), 1.9498(18), and 1.897(15) Å, respectively. The TiO_6_ octahedra are corner sharing and form a layer parallel to (0 0 1). Two of such layers are connected via common O1 oxygen atoms to form the perovskite-like layer with La^3+^ enclosed in the cavity between the two octahedral TiO_6_ layers (Figure 2c). Within the [Bi_1.45_La_0.55_O_2_]_2_ layer, cations are positioned alternatively above (Bi2 = 2h position) and below (Bi1 = 2g position with mixed occupancy of Bi^3+^ and La^3+^) the layer of the coplanar O4 oxygen atoms. Based on bond lengths, they may be regarded as bonded only to these O4 oxygen atoms with a Bi1–O4 atomic distance of 2.451(6) Å and a Bi2–O4 atomic distance of 2.201(4) Å. When considering the more distant anions, the Bi2-site is 4 + 4-fold coordinated with four bonds to oxygen atoms and four distant Bi2–Cl connections at 3.3557(6) Å. For the Bi1-site, the more distant Bi1–O3 bond length is 2.777(3) Å. A list of structural parameters (fractional atomic coordinated, equivalent isotropic and anisotropic atomic displacement parameters and occupation factors) is given in Tables S2–S4; full structural data are also available via the crystallographic information file (CIF).

Comparing the cell parameters of [LaTi_2_O_7_][Bi_1.45_La_0.55_O_2_]_2_Cl with cell parameters found in the literature for *n* = 2 Sillén–Aurivillius-type materials, e.g., Sr_2_Bi_3_Nb_2_O_11_Cl or Ba_2_Bi_3_Ta_2_O_11_Cl which are isostructural, it becomes evident that the cell parameters are significantly smaller (Table 1). This is likely due to the incorporation of smaller cations, Ti^4+^ (*r* = 0.61 Å) and La^3+^ (*r* = 1.36 Å), into the structure compared to Ta^5+^ (*r* = 0.64 Å)/Nb^5+^ (*r* = 0.64 Å) and Sr^2+^ (*r* = 1.44 Å)/Ba^2+^ (*r* = 1.61 Å).^59,60^ The refinement data of Nakada et al. show that also the partial substitution of Ta^5+^/Nb^5+^ with Ti^4+^ and Sr^2+^/Ba^2+^ with Bi^3+^ (*r* = 1.17 Å) already results in a reduction of the cell volume, however, less pronounced than in this work.^20^

**Table 1:** Cell Parameters of Several *n* = 2 Sillén–Aurivillius-Type Oxyhalides

compound	*a*/Å	*c*/Å	*V*/Å^3^	reference
La_2.1_Bi_2.9_Ti_2_O_11_Cl	3.86290(2)	17.98680(4)	268.44(1)	this work
Sr_2_Bi_3_Nb_2_O_11_Cl	3.910(9)	18.455(1)	282.27(2)	(20)
	3.914(9)	18.476(7)	283.18 (2)	(31)
Ba_2_Bi_3_Nb_2_O_11_Cl	3.968(3)	18.799(1)	296.05(3)	(20)
	3.969(7)	18.747(5)	295.43(2)	(31)
SrBi_4_TiNbO_11_Cl	3.878(8)	18.365(8)	276.32(1)	(20)
BaBi_4_TiNbO_11_Cl	3.916(6)	18.692(2)	286.00(1)	(20)
Ba_2_Bi_3_Ta_2_O_11_Cl	3.960(7)	18.789(1)	294.75(1)	(20)
SrBi_4_TiTaO_11_Cl	3.885(6)	18.357(1)	277.15(3)	(20)
BaBi_4_TiTaO_11_Cl	3.912(2)	18.724(3)	286.58(1)	(20)

**Figure 4:**
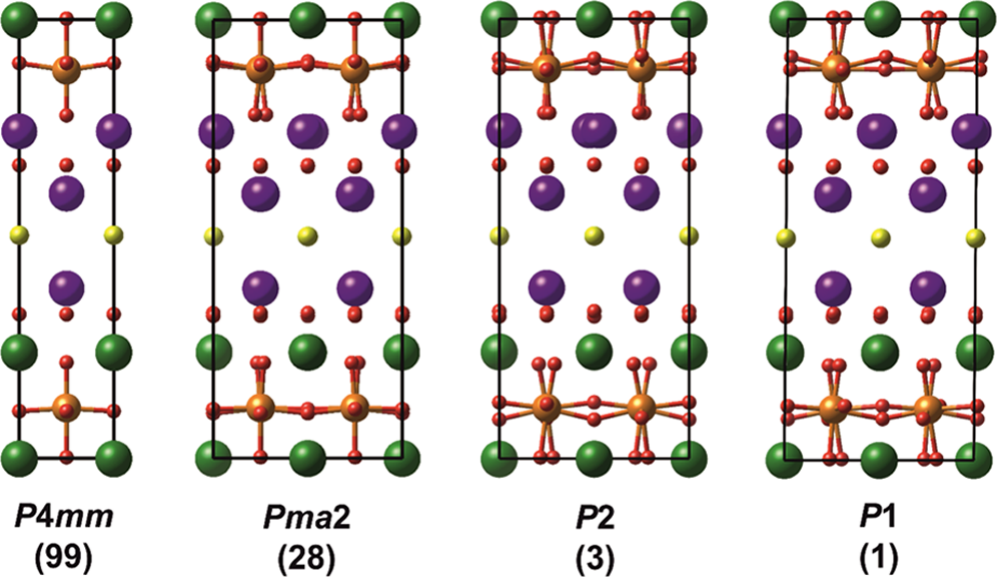
DFT-computed structures starting from the high-symmetry *P*4**mm** space group (on the left) and introducing modulations corresponding to unstable phonon modes until a stable structure with *P*1 symmetry (on the right) is obtained. Bi positions are labeled in violet, Ti in orange, Cl in yellow, O in red, and La in green.

**Figure 5:**
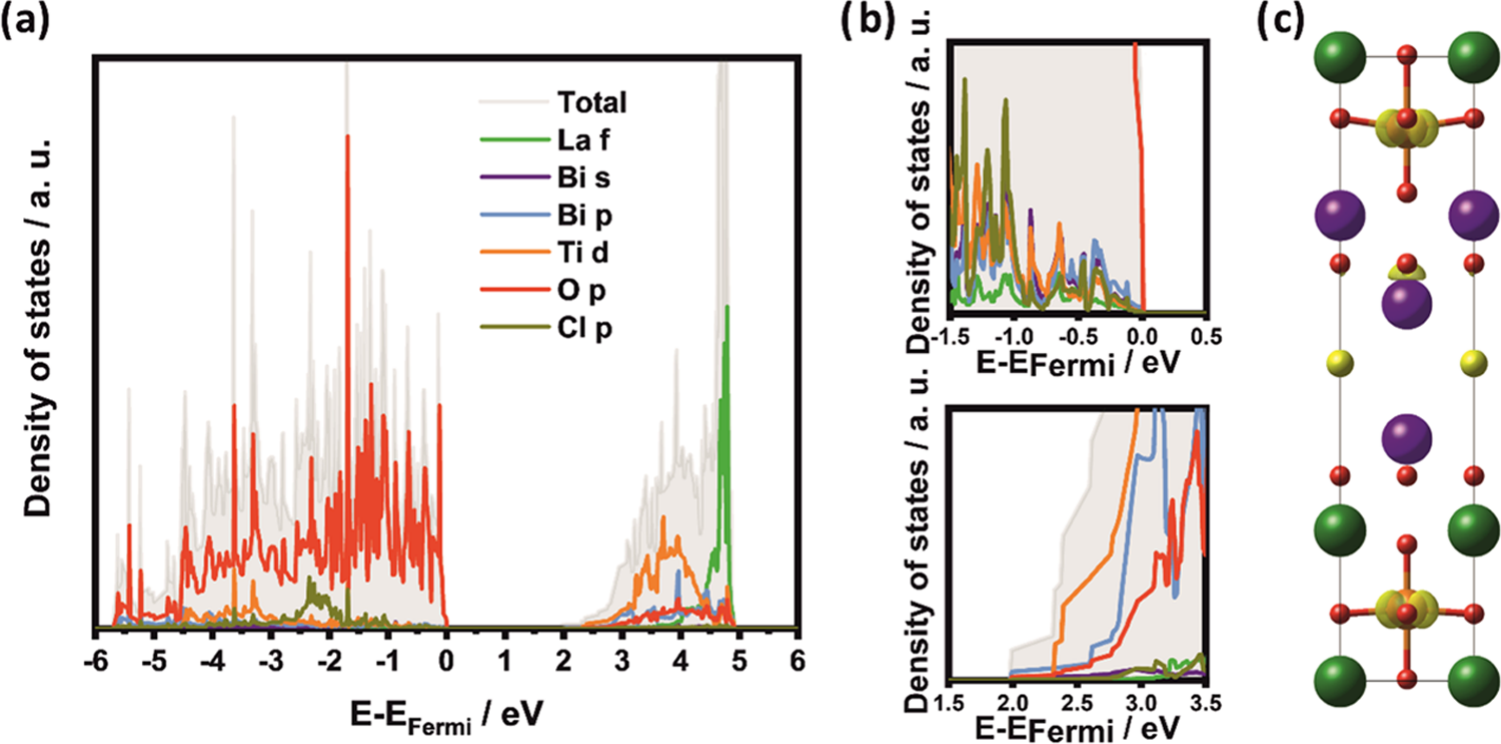
Main orbital-resolved contributions to the density of states (a), close-up of VBE (top) and CBE (bottom) (b), and integrated local density of states (0.002 e/Å^3^ isosurface) in an energy range from the CBE to CBE +0.5 eV using the high-symmetry *P*4**mm** space group (c).

In order to further understand the structure, DFT calculations are performed. The unit cell used as the starting point for the DFT calculations is the **P**4**mm** unit cell (Figure 4), which is a cation-ordered derivative of Sr_2_Bi_3_Nb_2_O_11_Cl (ICSD-196066) with one layer adjacent to the perovskite block being purely Bi^3+^ and the other purely La^3+^.^31^ This choice is motivated by La showing a strong preference to reside close to the perovskite layer as interchanging positions with Bi from the rock-salt layer will raise the energy by 1.30 eV. This also explains the XRD finding that only the Bi^3+^ (Bi2) position close to the perovskite layer is partially substituted by La^3+^. Using a cation-ordered cell with a consequently reduced symmetry compared to the experimental *P*4/*mmm* space group is necessary within our DFT setup that does not allow for partial occupations. Larger cells that could better reflect the experimental stoichiometry would lead to computationally intractable phonon calculations. The fully relaxed **P**4**mm** unit cell contains nine imaginary (unstable) phonon modes, two at the zone center, one at (1/2, 0, 0), and six at (1/2, 1/2, 0), which lead to a maximal energy lowering of 0.21 eV per unit cell. Sequentially following the most energy-lowering instability and redetermining phonons reduces the symmetry from **P**4**mm** to *Pma*2, *P*2, and ultimately *P*1. As shown in Figure 5, these distortions are mainly associated with displacements of oxygen and Bi atoms from their high-symmetry positions, combining to the usual octahedral rotations occurring in perovskites. The large displacements of oxygen atoms in the perovskite layer and of Bi in the perovskite-adjacent layer are in excellent agreement with the anisotropy determined for the crystal structure refined in the *P*4/*mmm* space group. We note that these distortions decrease the bond overlap and hence the band dispersion and therefore increase the DFT-PBEsol band gap from 1.99 eV in the **P**4**mm** space group to 2.17 eV in the dynamically stable *P*1 space group.

In conclusion, based on the results of X-ray diffraction studies, the structure can be described as an Sillén–Aurivillius-type intergrowth structure with *P*4/*mmm* as the space group, similar to Sr_2_Bi_3_Nb_2_O_11_Cl (ICSD-196006) reported by Charkin et al.^31^ These structures are related to Aurivillius phases, which consist of n = perovskite layers [A_*n*–1_B*_n_*O_3*n*+1_] with A = 12-fold and B = 6-fold coordinated cation, sandwiched by [Bi_2_O_2_]^2+^ layers, however, containing additional halide layers.^32^ From the structure refinements, we obtain a double-layered (*n* = 2) Sillén–Aurivillius-type structure with a chemical composition of [LaTi_2_O_7_][Bi_1.45_La_0.55_O_2_]_2_Cl or La_2.1_Bi_2.9_Ti_2_O_11_Cl. Compared to the target compound, the synthesis product is Bi-deficient. Comparing the compound composition determined by XRD pattern refinement with the atomic ratios based on EDX analysis, it becomes apparent that the atomic ratios of the cations (La/Ti: 1.09, Bi/Ti: 1.40) based on SEM-EDX data correspond very well with the atomic ratios of the composition La_2.1_Bi_2.9_Ti_2_O_11_Cl determined from structure refinement (La/Ti: 1.05, Bi/Ti: 1.45). The resulting partial substitution of La^3+^ on the Bi2-site with a site occupancy of 0.55 La^3+^ and 0.45 Bi^3+^ could be the reason for the superstructure observed by SAED TEM, if there is partial order. This Bi deficiency can be explained by the fact that Bi is volatile and easily lost during solid-state synthesis at elevated temperatures. Our DFT calculations predict that substituting 1/3 of Bi by La in the most favorable perovskite-adjacent layers will increase the DFT-PBEsol band gap by 0.19 eV. This indicates that the stoichiometric compounds could be even more suitable for visible light absorption. However, tying to increase the Bi-content in the compound toward the stoichiometric ratio by adding a Bi precursor in excess did not result in phase pure compounds.

### Morphology and Crystallinity

SEM studies show that La_2.1_Bi_2.9_Ti_2_O_11_Cl forms square-shaped plate-like particles with an average particle size of 1.8 ± 0.6 μm (measured along the particle edge) and an average thickness of 138 ± 45 nm. The particle size distribution is presented in Figure S4. SEM and TEM imaging (Figure 3a,d) where the scale bar in both cases corresponds to 1 μm indicates that no pores are present, which is common for flux-assisted syntheses and results in a low specific surface area of around 2 m^2^ g^–1^ (Figure S5).^61^ This specific surface area is in the same range as most of the double-layered Sillén–Aurivillius-type compounds synthesized by Nakada et al.^20^ Bright field TEM imaging and SAED patterns (Figure 3b) reveal that the particles are single crystalline. As mentioned above, the SAED pattern can be assigned to the tetragonal *P*4/*mmm* structure viewed along the [001] direction. The side facets belong to the {100} family as schematically depicted in Figure 3c. The anisotropic plate-like growth is a prominent feature of the Aurivillius and Sillén phases.^60,62^ Flux methods allow powder particles to grow into their thermodynamic equilibrium shape (or close to it), the plate-like shape with a short axis in the [001] direction being indicative of a low surface energy of the (001) facets.^63^

### Optical Properties and Electronic Structure

**Figure 6:**
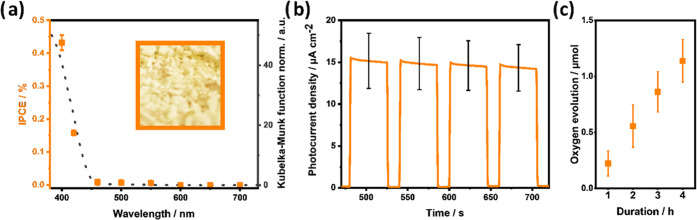
Kubelka–Munk function obtained from UV–vis diffuse reflectance measurements (dashed line) and IPCEs (filled squares) (a). The inset in panel (a) displays a photo of the oxyhalide powder. Results of photoelectrochemical (b) and photocatalytic measurements (c).

By eye, La_2.1_Bi_2.9_Ti_2_O_11_Cl appears slightly yellow (inset Figure 6a), therefore indicating an absorption in the visible light range. This assumption is confirmed by UV–vis diffuse reflectance spectroscopy (Figure 6a) indicating a band gap of 2.8 eV (443 nm) determined by extrapolation (Figure S6). Electronic structure calculations can help to uncover the origin of this rather small band gap.

The DFT-computed electronic structure (Figure 5a,b) reveals no Cl contribution close to the VBE, which is typical for Sillén–Aurivillius phases.^13,19−21,64^ However, a marked tail lowers the CBE. The integrated local density of states of this tail can be partially associated with Bi 6p states in the rock-salt layer, i.e., the Bi2-site adjacent to the Bi-containing perovskite layer (Figure 5c). Ti 3d (t_2g_) states located close to the CBE also contribute to this integrated local density of states. Nevertheless, these states are located at higher energies than the Bi 6p-derived bands. Hence, it seems sensible to assign a band gap lowering effect to Bi located adjacent to the perovskite layer. These observations about the CBE formation correspond well with DFT band structures of single-, double-, and multilayered Sillén–Aurivillius-type oxyhalides where the CBE is mainly composed of Bi 6p orbitals, and the d^0^ transition-metal orbitals contribute to the conduction band only at higher energies.^13,20−22^ Mentionable is a contribution of Bi 6s to the valence band primarily at lower levels (Figure S7), with small contributions due to covalent Bi–O bonds around the VBE, which is similar to previous reports.^20,21^ However, since the main contribution of these orbitals are positioned at much lower energies than the VBE, their influence on the optical properties is assumed to be negligible as also suggested by Kato et al.^16^

In accordance with the theoretical results, which predict a similar electronic structure as in other Sillén–Aurivillius-type oxyhalides, the 2.80 eV band gap of La_2.1_Bi_2.9_Ti_2_O_11_Cl is in the same range as Nb- and Ta-based double-layered Sillén–Aurivillius-type oxyhalides, e.g., for A′_2_Bi_3_Ta_2_O_11_Cl (A′ = Ba, Sr, Pb), the band gap varies between 2.86 and 2.57 eV.^20^ However, the A′_2_Bi_3_Ta_2_O_11_Cl compounds with partial B-site substitution of Ta^5+^ by Ti^4+^ reported in the same study exhibit smaller band gaps in the range of 2.48–2.60 eV. In order to compensate the alivalent substitution of Ti^4+^ on a Ta^5+^-site, Nakada et al. cosubstituted Bi^3+^ on the A-site. The authors suggested that the increased Bi-content in comparison to the unsubstituted compounds reduced the band gap. This is very plausible considering that the influence of the B-site cation on the CBE is limited in Sillén–Aurivillius-type compounds.^21^ In the present work, the Bi-content is slightly lower than in the unsubstituted A′_2_Bi_3_B_2_O_11_Cl compounds (A′ = Sr, Ba; B = Ta, Nb) and in consequence even lower with respect to the Ti-substituted compounds reported by Nakada et al. Therefore, it is sensible that La_2.1_Bi_2.9_Ti_2_O_11_Cl exhibits a similar or even slightly larger band gap than Ta- and Nb-based double-layered compounds.^20,21^ The reduced band gap of La_2.1_Bi_2.9_Ti_2_O_11_Cl, compared to, for example, La_2_Ti_2_O_7_ (3.9 eV) or Bi_2_Ti_2_O_7_ (2.95 eV), is therefore linked to the contribution of Bi 6p orbitals located at the Bi2-site, which shift the CBE as also derived from our DFT calculations.

### Photocatalytic and Photoelectrochemical Activity

After preparation of particle-based electrodes with a layer thickness of about 3 ± 0.7 μm, linear scan voltammetry measurements are performed in voltage ranges from 0 to 0.7 and 0.7 to 1.23 V vs RHE, where the activity toward the hydrogen and the oxygen evolution reaction is assessed, respectively (Figure S8). Mentionable is that a small cathodic photocurrent is observable at potentials below 0.3 V vs RHE (Figure S8b), which indicates that the material is active for the hydrogen evolution reaction. However, this is true to a much lower extent than for the oxygen evolution reaction (Figure S8a). Based on the much stronger activity for the oxygen evolution reaction, it is assumed that the material shows n-type semiconducting character, which has also been observed for other double-layered Sillén–Aurivillius-type oxyhalides.^20^

Chronoamperometry under visible light illumination AM 1.5G (100 mW cm^–2^ with a UV-cutoff filter (λ > 420 nm)) was performed at 1.2 V vs RHE (Figure 6b) probing the visible light activity toward the oxygen evolution of the compound. The influence of the pH value on the performance of the electrolyte was also investigated (Figure S9) by measuring the photocurrent density at pH 2.26, pH 7, and pH 13.34. We obtain the best performance under acidic conditions. Using 0.1 M Na_2_SO_4_ (pH = 2.26) as an electrolyte, La_2.1_Bi_2.9_Ti_2_O_11_Cl synthesized at 820 °C for 20 h resulted in a photocurrent density of about 15 μA cm^–2^ (Figure S10), while all other tested synthesis condition combinations (20 h at 780 °C, and 800 °C and 5 and 10 h at 820 °C) resulted in compounds with lower photoelectrochemical activity. The photocurrent density is relatively stable compared to other visible light-driven photoelectrodes based on mixed anion materials, which show considerable decline after several seconds up to a few minutes.^65−68^ The stability of La_2.1_Bi_2.9_Ti_2_O_11_Cl is likely to originate from its band structure. In contrast to oxynitrides, oxysulfides, and oxyhalides where the VBE is composed mainly of the non-oxide anion p orbitals (e.g., N 2p or Cl 3p), the VBE of this material is dominated by O 2p orbitals as discussed prior. This allows stable water splitting since photogenerated holes at the VBE can efficiently oxidize water instead of oxidizing the non-oxide anions, which would result in photocorrosion or photooxidation and a decrease of the photocatalyst stability.^69^ Compared to photocurrent densities of unsubstituted Nb- and Ta-based double-layered Sillén–Aurivillius compounds measured in 0.1 M Na_2_SO_4_ at a pH of 6.8 by Nakada et al., the observed photocurrent density at 1.2 V vs RHE is higher in this work. Comparable performance values are measured for the Ti-substituted double-layered Ta-based oxyhalides with the composition of BaBi_4_TiTaO_11_Cl and SrBi_4_TiTaO_11_Cl. The authors suggest that the enhanced photoelectrochemical performance of these compounds is related to the increased Bi-content and in consequence reduced band gap compared to the unsubstituted materials Ba_2_Bi_3_Ta_2_O_11_Cl and Sr_2_Bi_3_Ta_2_O_11_Cl.^20^ Indeed, the electronic structure calculations of the present work confirm that enhancing the Bi-content improves the visible light activity and therefore the photocatalytic performance of double-layered Sillén–Aurivillius compounds. However, to rationalize the observed performance improvement of this work with respect to the unsubstituted Ta-based compounds, the effect cannot be argued with the incorporation of Bi since the Bi-content and the band gap of La_2.1_Bi_2.9_Ti_2_O_11_Cl is in the same range as for Ba_2_Bi_3_Ta_2_O_11_Cl or Sr_2_Bi_3_Ta_2_O_11_Cl. Therefore, the higher photocurrent of La_2.1_Bi_2.9_Ti_2_O_11_Cl cannot be explained by improved light absorption as suggested for BaBi_4_TiTaO_11_Cl and SrBi_4_TiTaO_11_Cl. It rather indicates that the Ti^4+^-containing perovskite layer contributes positively to the catalytic performance of the oxyhalide material. In fact, Ozaki et al. underline the influence of the perovskite layer on the photocatalytic activity showing that the thickness of the perovskite layer has a significant impact.^22^ The positive influence of the Ti^4+^-containing perovskite layer is not surprising since Ti-based perovskite-related materials such as SrTiO_3_ or LaTiO_2_N show excellent photocatalytic activity.^35,70^ The additional incorporation of the trivalent La is likely to stabilize the Ti^4+^-containing perovskite layer.^37^ Consequently, the improved photocatalytic performance of La_2.1_Bi_2.9_Ti_2_O_11_Cl compared to Ta- and Nb-based oxyhalides is linked to an overlap of light absorption (narrow band gap) and catalytic activity effects (Ti-sites in the perovskite layer). In summary, La_2.1_Bi_2.9_Ti_2_O_11_Cl shows best-in-class photoelectrochemical performance comparable only to BaBi_4_TiTaO_11_Cl and SrBi_4_TiTaO_11_Cl.

In order to assess the wavelength dependence of the efficiency, IPCEs are measured (Figure 6a). The performance increase observed for wavelengths shorter than 450 nm agrees well with the absorption edge of La_2.1_Bi_2.9_Ti_2_O_11_Cl determined by UV–vis–reflectance spectroscopy (443 nm). In the literature, no IPCE measurements have been reported so far for double-layered Sillén–Aurivillius-type oxyhalides. These measurements demonstrate the visible light sensitivity. However, with IPCE below 1% at 400 nm, the material needs to be improved to be suitable for practical applications. In addition, the photocatalytic activity toward the oxygen evolution reaction is measured in the presence of a sacrificial agent (5 mM FeCl_3_) under visible light irradiation. The photocatalytic performance remains low with a value of around 1.2 μmol of oxygen after 4 h. In the publication of Nakada et al. similar values are determined for Ti^4+^-containing compounds.^20^ For La_2.1_Bi_2.9_Ti_2_O_11_Cl, no hydrogen evolution could be quantified via gas chromatography during photocatalytic measurements in the presence of a sacrificial agent (methanol) either with or without a UV-cutoff filter even after applying Pt nanoparticles as a cocatalyst. These results indicate that the activity toward the hydrogen evolution reaction requires application of a bias, which is not the case for other double-layered Sillén–Aurivillius-type oxyhalides such as Ba_2_Bi_3_Nb_2_O_11_Cl.^20^

## Conclusions

In this work, we demonstrate the fabrication of a novel member of the layered Sillén–Aurivillius-type family [A_*n*–1_B*_n_*O_3*n*+1_][Bi_2_O_2_]_2_X*_m_* by a facile one-step solid-state flux synthesis. To the best of our knowledge, the double-layered (*n* = 2) Sillén–Aurivillius-type compound A′_1+*x*_A_4–*x*_B_2_O_11_Cl (with A′ = La, A = Bi, B = Ti) has not been reported before. The structure and composition of the title compound is thoroughly characterized based on XRD pattern refinement, SEM and TEM EDX, and DFT calculations. Based on the results of X-ray diffraction studies, the structure can be described as a Sillén–Aurivillius-type intergrowth structure with the space group *P*4/*mmm*. From the structure refinements, we obtain a double-layered (*n* = 2) Sillén–Aurivillius-type structure with a chemical composition [LaTi_2_O_7_][Bi_1.45_La_0.55_O_2_]_2_Cl or La_2.1_Bi_2.9_Ti_2_O_11_Cl. The atomic ratios of the cations ascertained by XRD pattern refinement correspond very well with the atomic ratios determined by EDX analysis. Due to the incorporation of smaller cations, the cell volume decreases compared to Nb- and Ta-based double-layered Sillén–Aurivillius oxyhalides. The electronic structure is similar to other double-layered Sillén–Aurivillius-type oxyhalides where the VBE is predominantly formed by O 2p orbitals, and the CBE is defined by Bi 6p orbitals. The narrow band gap of 2.8 eV provides visible light sensitivity and is mainly associated with the contribution of Bi 6p orbitals to the CBE that are mainly located at the Bi2-site where Bi is adjacent to the perovskite layer. By integrating this compound as an active material on photoelectrodes, the observed stable photoelectrochemical performance (15 μA cm^–2^) as a photoanode is higher than for unsubstituted Ta- or Nb-based double-layered Sillén–Aurivillius-type oxyhalides and similar to the ones partially substituted with Ti^4+^. Compared to unsubstituted Ta- and Nb-based compounds, this performance boost is probably related to the presence of Ti^4+^ species in the perovskite slab. The n-type semiconducting material exhibited photocatalytic activity toward the oxygen evolution reaction under visible light illumination (0.3 μmol h^–1^) but is inactive for the hydrogen evolution reaction.

This work underlines the potential of composition variation in Sillén–Aurivillius-type compounds such as the incorporation of other trivalent cations on the A-site. It emphasizes that the nature of the A-site cation adjacent to the perovskite layer exhibits a more pronounced influence on the band gap than other A-site positions. Hence, targeting this particular position, for example, with Bi substitution should enable a further increase of the visible light activity of Sillén–Aurivillius-type compounds. Advanced characterization techniques like neutron diffraction could be employed to explore the complex site occupancy and symmetry relations in these compounds further. This work also shows that the concept of changing the composition of the perovskite layer, specifically the incorporation of Ti^4+^, has a beneficial influence on the photocatalytic activity. These concepts could be useful to further increase the photocatalytic or photoelectrochemical efficiency of Sillén–Aurivillius-type compounds, enabling stable solar water splitting under visible light illumination.

## Supporting Information Available

Supplementary experimental characterizations regarding the refinement structure, SEM-EDX mapping, SEM- and STEM-EDX quantitative data given in atomic ratios, evaluation of SAED diffraction pattern, SEM images and particle distribution, physisorption isotherms, UV–vis data evaluation, orbital-resolved DOS at lower energies, linear scan voltammetry measurements, pH dependence of photoelectrochemical performance and photoresponse for the hydrogen evolution reaction, and influence of synthesis conditions on photoresponse (PDF)
