# Supplementary material for: Synthesis and Structure of the Double-Layered Sillén–Aurivillius Perovskite Oxychloride La2.1Bi2.9Ti2O11Cl as a Potential Photocatalyst for Stable Visible Light Solar Water Splitting
Source: Inorg Chem. 2023 Apr 20;62(17):6649–60. doi: 10.1021/acs.inorgchem.3c00116 (PMC10155181; doi:10.1021/acs.inorgchem.3c00116)
Supplement: Supplementary file 1 — ic3c00116_si_001.pdf [file ic3c00116_si_001.pdf]

Supporting Information

Synthesis and structure of the double-layered Sillén-Aurivillius perovskite oxychloride $La_{2.1}Bi_{2.9}Ti_2O_{11}Cl$ as a potential photocatalyst for stable visible-light solar water splitting

*Valérie Werner†, Ulrich Aschauer‡, Günther J. Redhammer†, Jürgen Schoiber†, Gregor A. Zickler†, and Simone Pokrant†**

†Department of Chemistry and Physics of Materials, University of Salzburg, Jakob-Haringer-Str. 2A, 5020 Salzburg, Austria; ‡Department of Chemistry, Biochemistry and Pharmaceutical Science, University of Bern, Freiestraße 3, 3012 Bern, Switzerland

*simone.pokrant@plus.ac.at

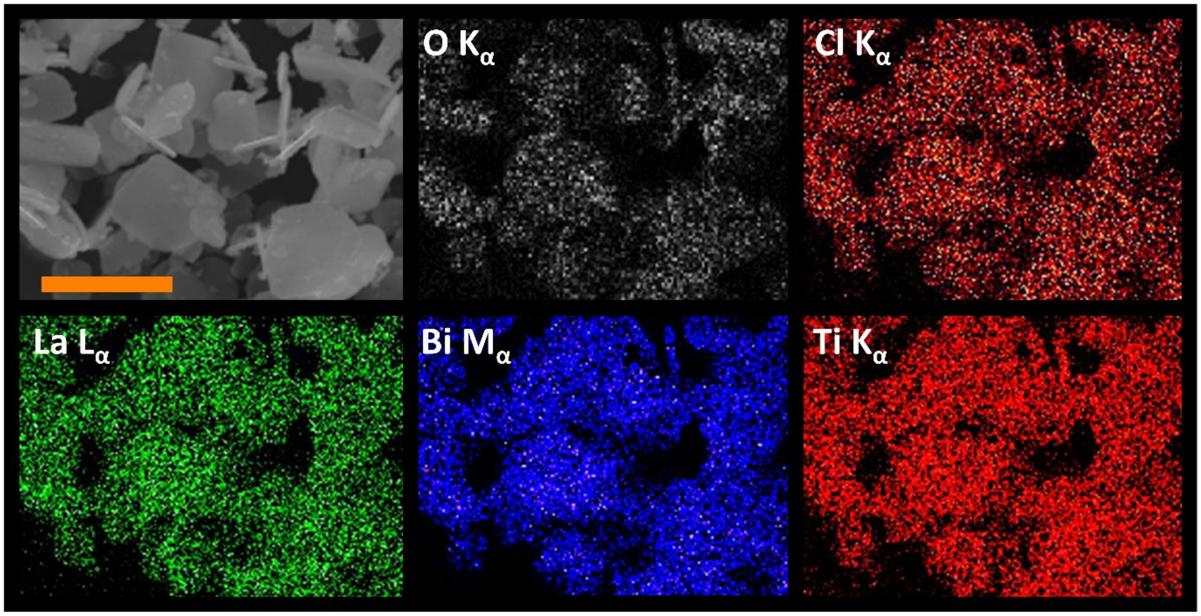

Figure S1. SEM-EDX mapping of $La_{2.1}Bi_{2.9}Ti_2O_{11}Cl$ particles. The scale bar corresponds to 4 μm.

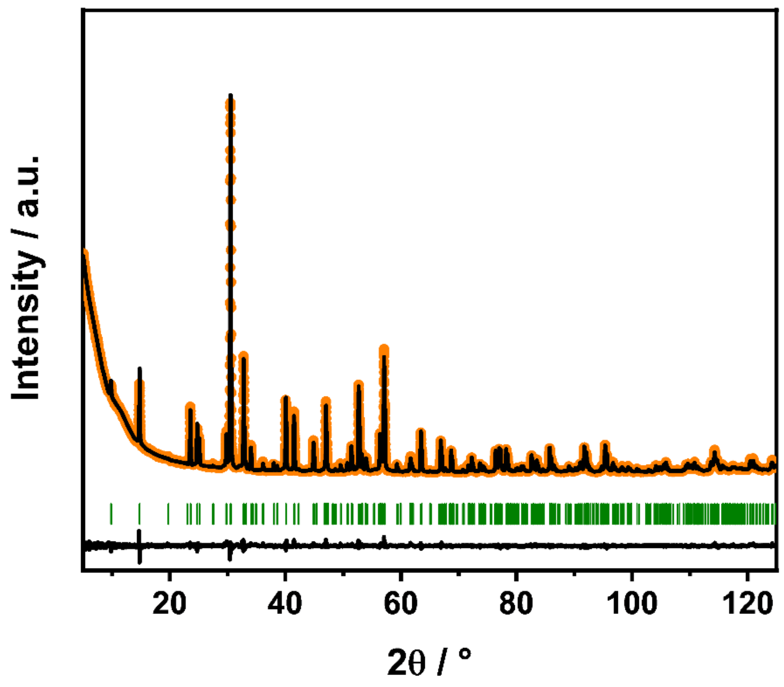

Figure S2. Laboratory XRD diffraction results. XRD pattern and structure refinement fit.

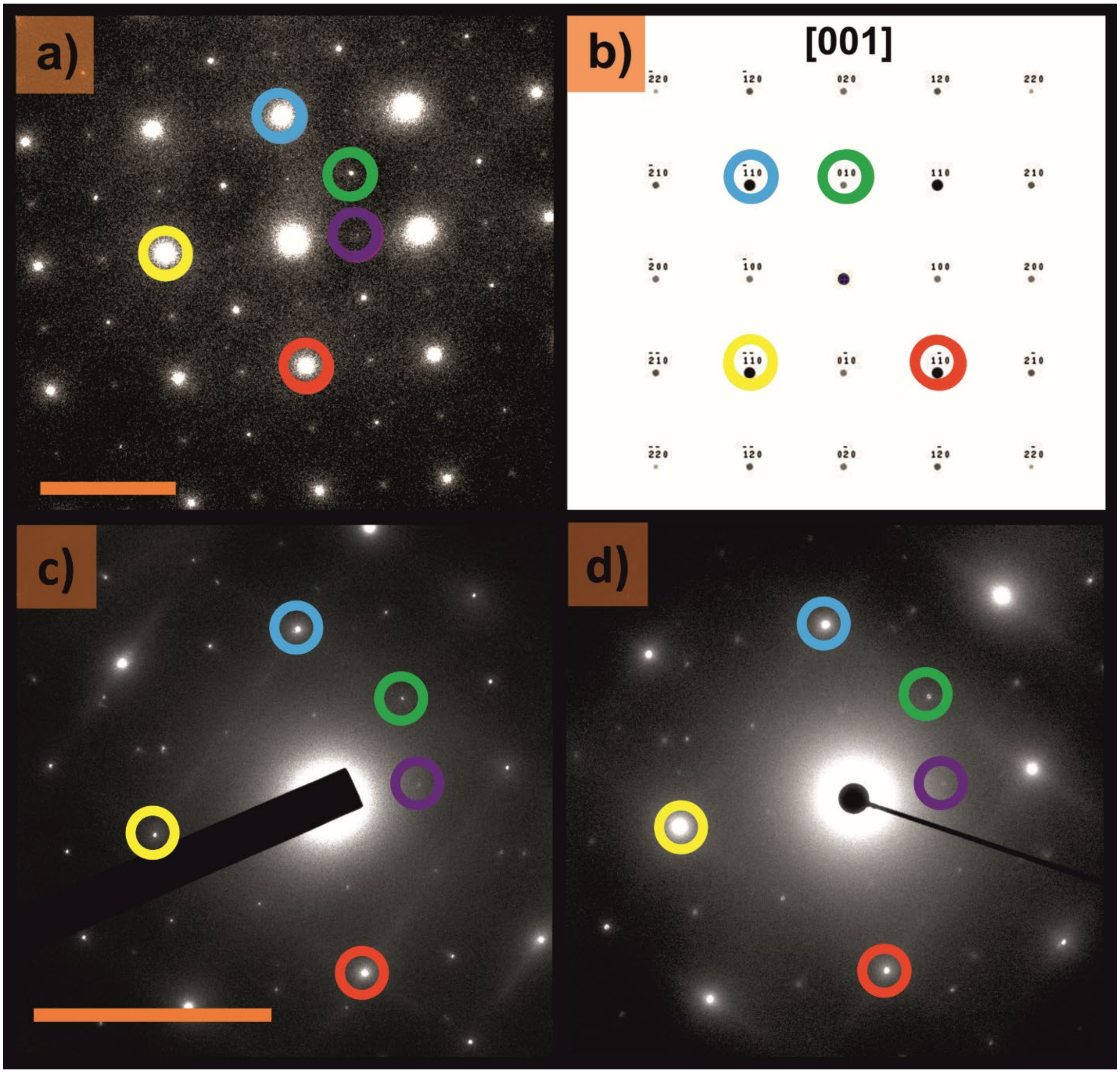

Figure S3. SAED pattern of a $La_{2.1}Bi_{2.9}Ti_2O_{11}Cl$ particle (a) and corresponding simulated pattern (b) using the unit cell determined by XRD in this work viewing along the [001] direction of the tetragonal *P*4*/mmm* structure.[1] The scale bar corresponds to 10/nm. The diffraction pattern simulation was performed with the JEMS software.[2] The violet circle indicates the position of an additional reflection (½, ½, 0). Similar reflections can be found in symmetry equivalent positions SAED diffraction patterns of a $La_{2.1}Bi_{2.9}Ti_2O_{11}Cl$ at 5° tilt in α (c) and β (d) direction are shown (scale bar 5/nm), indicating that the nature of the additional reflection at (½, ½, 0) might be kinematic.

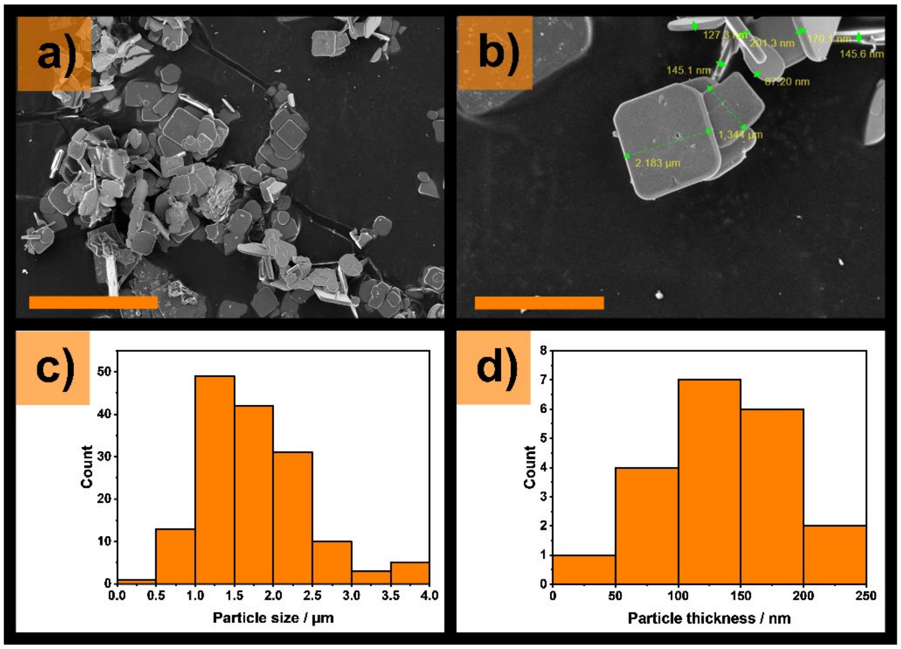

Figure S4. Representative SEM images ((a) and (b)) and particle-size and -thickness distribution determined by SEM image analysis ((c) and (d)). The scale bar in the SEM images corresponds to 12 µm in (a) and 3 µm in (b).

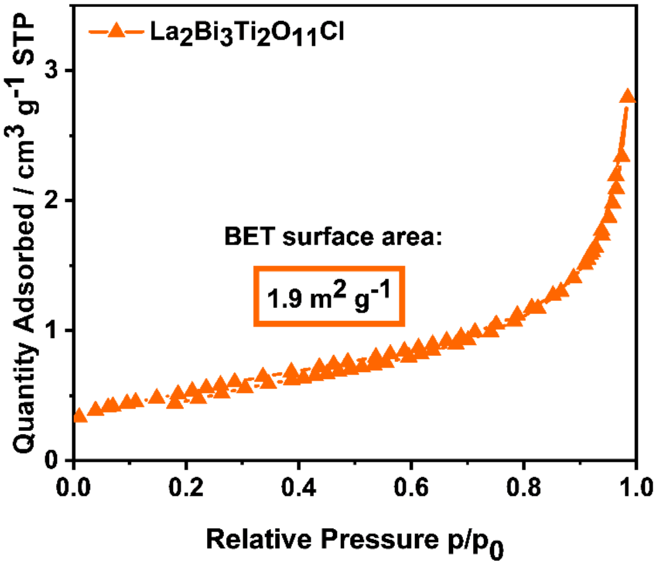

Figure S5. Nitrogen physisorption isotherms of $La_{2.1}Bi_{2.9}Ti_2O_{11}Cl$.

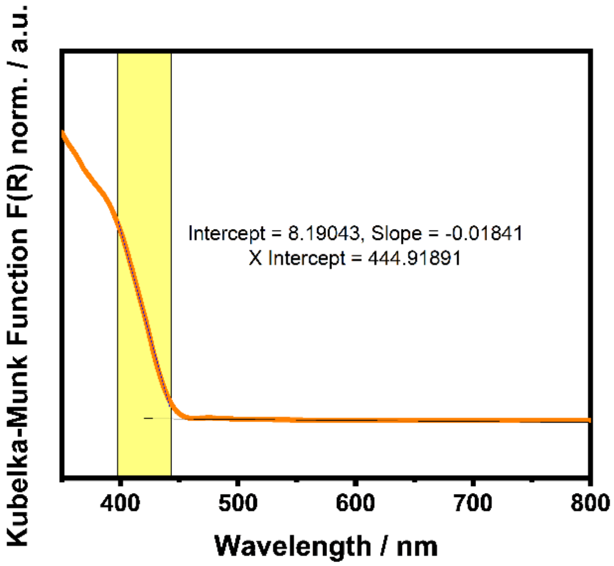

Figure S6. Determination of absorption edge via extrapolation of the Kubelka-Munk-function.

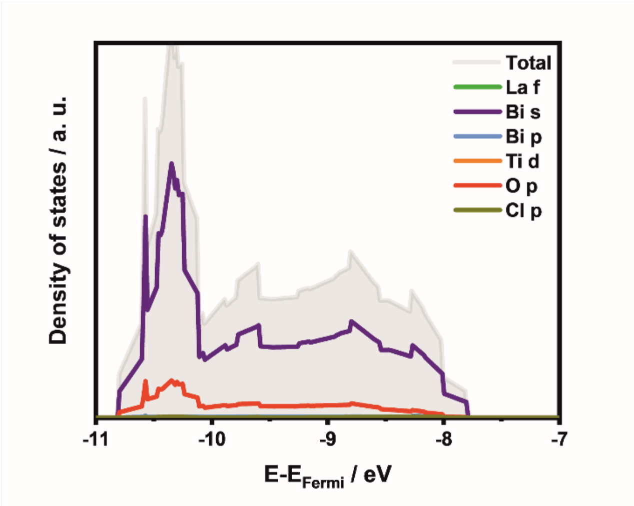

Figure S7. Close-up of orbital-resolved density of states at lower energies determined by DFT calculations.

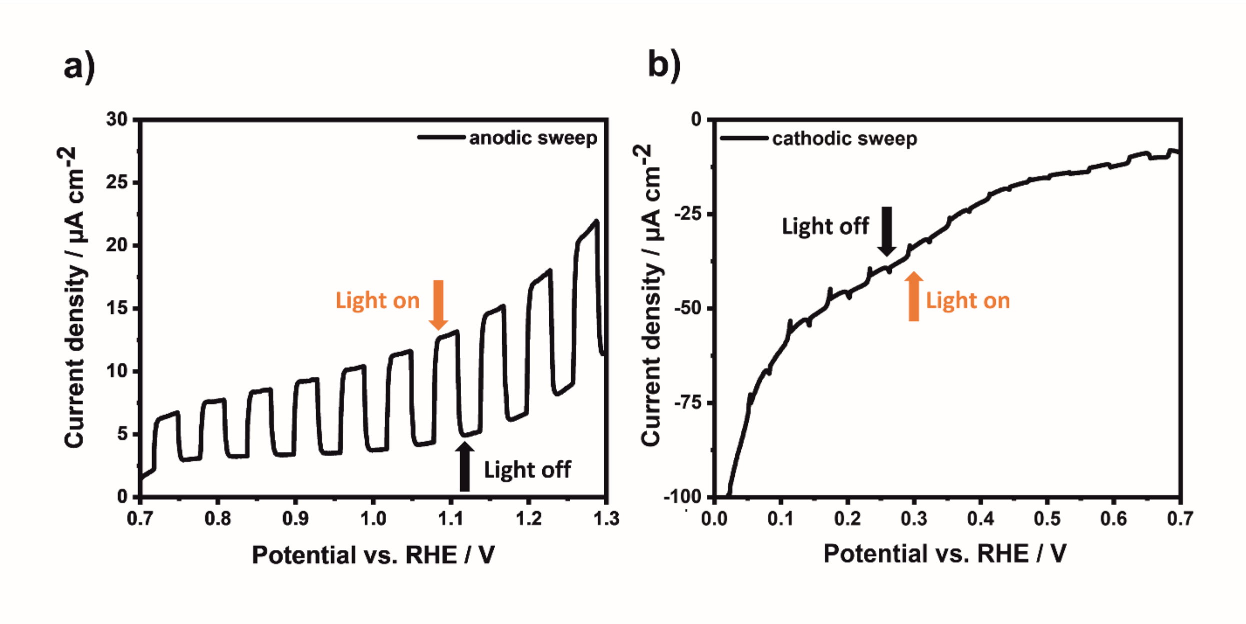

Figure S8. Linear scan voltammetry measurements (anodic sweep (a) and cathodic sweep (b)).

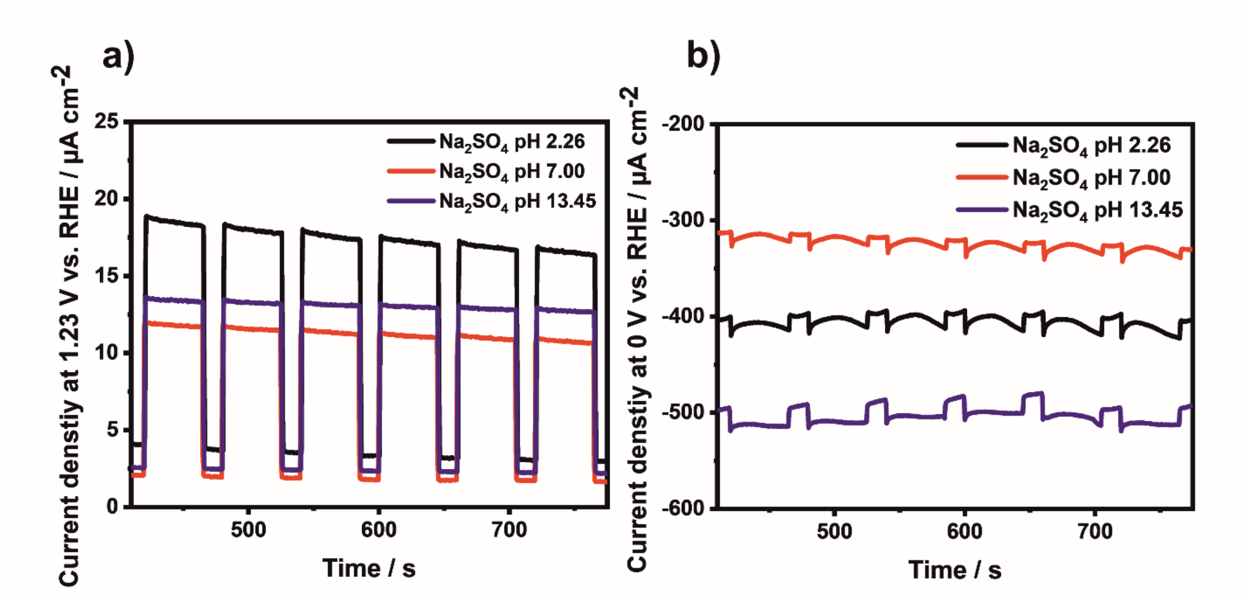

Figure S9. Influence of pH value on the photoelectrochemical performance and photoresponse towards the oxygen (a) and hydrogen (b) evolution reaction of a $La_{2.1}Bi_{2.9}Ti_2O_{11}Cl$ photoelectrode. The graphs show chronoamperometric measurements performed at 0 V vs. RHE and 1.23 V vs. RHE for t = 400 s to t = 800 s. These extracts were chosen for better visualization of the differences.

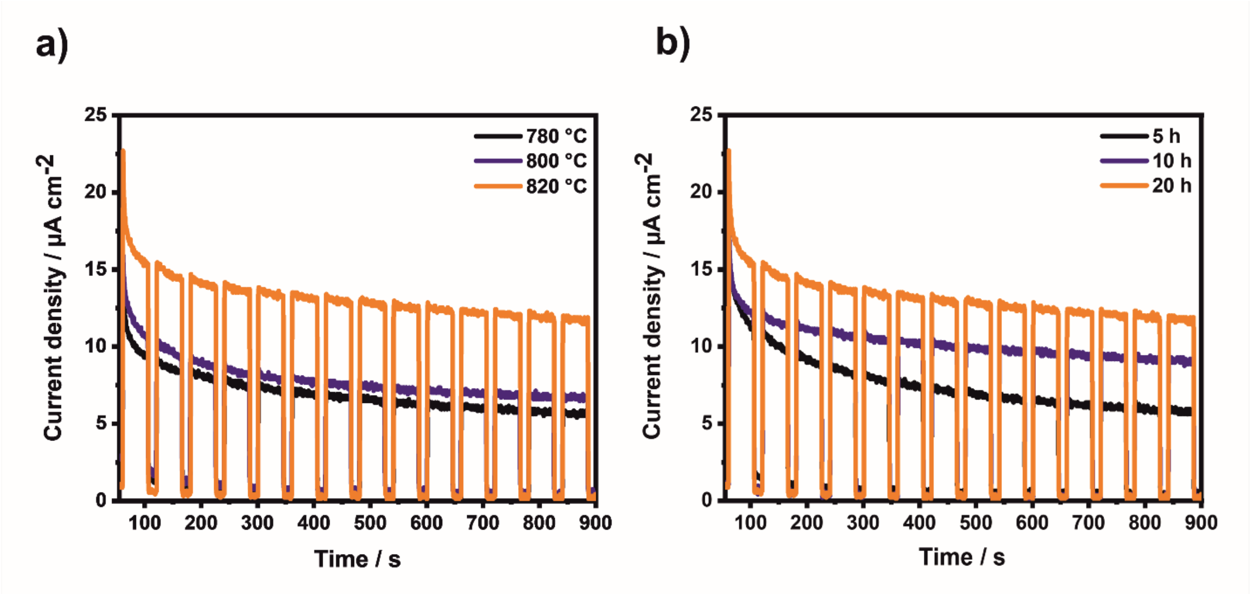

Figure S10. Chopped chronoamperometic measurements: Influence of synthesis condition by varying the temperature (duration 20 h) (a) and the duration at 820 °C (b)) on the photoresponse for the oxygen evolution reaction.

Table S1. Atomic ratios based on SEM-EDX analysis.

Elemental ratio	*Theoretical ratio*	*Ratios based on XRD refinement*	*SEM-EDX*	*Error*
Cl/Bi	0.33	0.36	0.29	0.02
Bi/Ti	1.50	1.45	1.40	0.05
La/Ti	1.00	1.05	1.09	0.05

Note: To obtain semi-quantitative information by relative quantification, the O Kα, Cl Kα, Bi Mα, La Lα and the Ti Kα edges were analyzed using the INCA software. The EDX detector signal was integrated and converted to ratios by a software routine which relies on the database of standards provided by the manufacturer (Oxford Instruments). The O/Cl ratio was not determined by SEM-EDX since the error bars for the determination of the light elements are high. The accuracy and precision of the critical La/Ti element ratio was ensured by calibrating the detector using copper and repeating the measurement at four sample sites.

Table S2. Structural parameters for $[LaTi_2O_7][Bi_{1.45}La_{0.55}O_2]_2Cl$, obtained from refinements of high resolution synchrotron X-ray diffraction data, λ= 0.6053 Å, d_{min} = 0.56 Å, Space group *P*4/*mmm* (# 123), *a* = 3.86290(2) Å, *c* = 17.98680(14) Å, Z = 4. Conventional Rietveld reliability R-factors (all data): R_p = 7.77%, R_{wp} = 7.71%, R_{Bragg} = 3.53%.

Site	*Wyckoff letter*	*Element*	*x*	*y*	*z*	U_{equ}^{*}	*Occupancy*
La1	1*a*	La	0	0	0	0.0200(7)	1
Bi1	2*h*	Bi	½	½	0.39163(5)	0.0235(3)	1
Bi2	2*g*	Bi	0	0	0.24899(9)	0.0435(7)	0.453(10)
La2	2*g*	La	0	0	0.24899(8)	0.0435(7)	0.547(10)
Ti1	2*h*	Ti	½	½	0.11555(18)	0.0155(12)	1
Cl1	1*b*	Cl	0	0	½	0.033(3)	1
O1	1*c*	O	½	½	0	0.074(8)	1
O2	4*i*	O	0	½	0.1006(7)	0.045(3)	1
O3	2*h*	O	½	½	0.2210(8)	0.049(5)	1
O4	4*i*	O	½	0	0.3329(5)	0.030(2)	1

* = equivalent isotropic atomic displacement parameter, calculated from anisotropic values for cations, given in Table S3, or isotropic value, refined directly for anions.

Note1: When refining the 2*g* Position with La only (full occupation) the R-values significantly increase to R_p = 26.02, R_{wp} = 24.70, R_{Bragg} = 15.74 %, when refining it with Bi only R_p = 13.45, R_{wp} = 12.94, R_{Bragg} = 6.88, thus giving strong evidence for the correctness of a mixed occupation of the 2*g* Position with Bi + La.

Note 2: Refining the structure with full anisotropic atomic displacement parameters for all atoms is also possible, yielding a slight improvement of Conventional Rietveld reliability R-factors (all data): R_p = 7.49%, R_{wp} = 7.59%, R_{Bragg} = 3.12%, results can be found under CSD entry # 2226811.

Table S3. Full set of anisotropic atomic displacement parameters for the structural data given in Table S2.

Site	U_{11}	U_{22}	U_{33}	U_{12}	U_{13}	U_{23}
La1	0.0215(6)	0.0215(6)	0.0169(9)	0	0	0
Bi1	0.0239(3)	0.0239(3)	0.0228(5)	0	0	0
Bi2	0.0324(5)	0.0324(5)	0.0656(9)	0	0	0
La2	0.0324(5)	0.0324(5)	0.0656(9)	0	0	0
Ti1	0.0172(11)	0.0172(11)	0.0123(14)	0	0	0

Table S4: Bond valence sums as calculated using the program BondStr (Fullprof Suite).

Atom	*Coordination*	*Average Distance*	*Bond Valence Sum*
La1	12	2.6741(17)	3.107(16)
Bi1	4	2.2040(22)	2.972(17)
Bi2	8	2.6132(15)	2.166(12)
La2	8	2.6132(15)	2.674(15)
Ti1	6	1.9618(23)	4.079(29)

ABBREVIATIONS

SAED, selected area electron diffraction, SEM-EDX, scanning electron microscopy-energy-dispersive X-ray spectroscopy, XRD, X-ray diffraction

REFERENCES

1. Charkin, D. O.; Akinfiev, V. S.; Alekseeva, A. M.; Batuk, M.; Abakumov, A. M.; Kazakov, S. M., Synthesis and cation distribution in the new bismuth oxyhalides with the Sillén–Aurivillius intergrowth structures. *Dalton Transactions* **2015,** *44* (47), 20568-20576.
2. Stadelmann, P. JEMS: Java Electron Microscopy Software. https://www.jems-swiss.ch/.
